# Arsenic trioxide-induced cardiotoxicity: the protective effect of 2-aminoethoxydiphenyl-borate: 2-APB alleviates ATO-induced cardiotoxicity

**DOI:** 10.3724/abbs.2024036

**Published:** 2024-03-18

**Authors:** Jia Feng, Ruimeng Tian, Guilin Lu, Wenjuan Qin

**Affiliations:** 1 Department of Ultrasonography People’s Hospital of Deyang City Deyang 618000 China; 2 Department of Ultrasonography the First Affiliated Hospital of Shihezi University Shihezi 832008 China

Arsenic trioxide (ATO) has been approved by the United States Food and Drug Administration as a treatment for acute promyelocytic leukemia (APL) [1]. Related studies have shown that ATO has a good therapeutic effect on some solid tumors, such as liver cancer and breast cancer [ 2, 3]. However, ATO has a toxic effect on the heart, which can manifest as chest tightness, palpitation or tachycardia, acute heart failure, torsade de pointes, ventricular fibrillation, and even death [4]. Therefore, the cardiotoxic side effects of ATO seriously limit its clinical application. Therefore, how to maximize the anti-tumor effect of ATO and minimize its cardiotoxicity has become an urgent problem for studying. Previous studies have shown that 2-aminoethoxydiphenyl borate (2-APB), a boron-containing compound with various biological activities, has protective effects on the heart [5]. Therefore, in this study we established ATO-induced cardiotoxicity models and 2-APB intervention therapy models and performed a preliminary study on alleviating ATO-induced cardiotoxicity.

Sprague-Dawley male rats (age: 12 weeks; grade: specific pathogen-free; weight: 180–200 g) were provided by Shandong Experimental Animal Center (production license number: SCXK-2020-0005) and randomly divided into five groups with 8 rats in each group: the control group (control), 2-APB negative control group (2-APB), natural recovery group after ATO poisoning (ATO), low-dose 2-APB intervention group after ATO poisoning (low-dose 2-APB), and high-dose 2-APB intervention group after ATO poisoning (high-dose 2-APB). First, a model of ATO poisoning was generated (ATO was injected daily for 10 days): the ATO, low-dose 2-APB, and high-dose 2-APB groups were intraperitoneally injected with 5 mg/kg/day ATO [6], and the control and 2-APB groups were intraperitoneally injected with the same volume of saline. Then, 2-APB was administered (2-APB was injected every other day for 21 days). After modelling, the rats in the low-dose 2-APB, high-dose 2-APB, and 2-APB groups were injected intraperitoneally with 2-APB at 2, 4, and 4 mg/kg/day 2-APB, respectively, and the control and ATO groups were injected with the same volume of saline. Throughout the experiment, the rats were kept in the animal room of Shihezi University (light-dark cycle: 12 h; humidity: 40%‒60%; temperature: 23±2°C) and fed with standard feed. At the end of drug administration, the rats were subjected to echocardiography to obtain speckle tracking echocardiography (STE) and myocardial contrast echocardiography (MCE) data. After echocardiography, blood and heart samples were collected from the rats in each group, after which oxidative stress-related enzymes were measured, myocardial myofibril structure was observed, and CD31 immunofluorescence staining was performed. All the experiments were carried out according to the experimental animal guidelines and procedures approved by the Experimental Animal Ethics Committee of the First Affiliated Hospital of Shihezi University (approval No: A2021-060-01). All data were analyzed using SPSS22.0 software. Measurement data with a normal distribution are expressed as the mean±standard deviation, and ANOVA analysis was used for comparisons among groups.

STE tracks all the spot displacements of myocardial tissue in the whole cardiac cycle; finally, it calculates the strain of each layer of the myocardium in all directions, which reflects the exercise ability of the myocardium [7]. Compared with that in the control group, the global longitudinal strain (GLS) of the left ventricle in the ATO group decreased, especially the GLS in the endocardium (GLS-endo). After intervention with different doses of 2-APB, the above parameters recovered to different degrees, which suggested that ATO caused damage to all layers of the myocardium, endocardial injury became more severe, and different doses of 2-APB restored myocardial function to different degrees. Among the above parameters, the recovery of GLS-endo was the most obvious. The possible reason for the above results is that the endocardial myocardium is supplied by the peripheral vessels of the coronary artery, the endocardial myocardial metabolism rate is fast, and it is very sensitive to changes in blood supply and oxygen supply. Once the oxygen supply or blood supply changes, the exercise ability of cardiomyocytes changes. The above results suggested that ATO can damage all layers of the myocardium, while 2-APB can restore the exercise ability of the myocardium ( Table 1).

**Table:** **Table 1** Comparison of GLS in different layers of the left ventricle and MCE-related parameters in the post-wall myocardium of rats in each group ( $\overset{¯}{x}$ ± *s*, *n*=8)

Group	Control	2-APB	ATO	Low-dose 2-APB	High-dose 2-APB	*F*	*P*
GLS-endo (%)	22.69±1.83	22.74±1.77	11.18±1.88 ^a^	14.99±2.14 ^ab^	18.10±2.21 ^abc^	51.355	0.000
GLS-mid (%)	16.36±1.19*	15.98±1.33*	8.40±1.13 ^a^*	10.94±1.79 ^ab^*	13.44±1.29 ^abc^*	49.060	0.000
GLS-epi (%)	10.38±1.09 ^#^	10.58±1.26 ^#^	5.61±0.58 ^a#^	5.75±0.83 ^a#^	7.35±0.68 ^abc#^	54.937	0.000
WIS (dB/s)	27.32±4.27	27.63±3.43	6.03±1.50 ^a^	10.63±3.46 ^ab^	15.90±3.02 ^abc^	71.321	0.000
PI (dB)	128.57±5.56	127.47±5.76	18.25±5.21 ^a^	45.39±4.58 ^ab^	78.07±3.58 ^abc^	769.598	0.000
AUC	2709.49±212.00	2720.85±184.19	422.20±175.26 ^a^	835.54±105.87 ^ab^	1291.34±184.60 ^abc^	294.056	0.000

^a^ *P*<0.05 compared with the control group; ^b^ *P*<0.05: compared with the ATO group; ^C^ *P*<0.05: compared with the low-dose 2-APB group; * *P*<0.05: compared with the same group of GLS-endo; ^#^ *P*<0.05: compared with the same group of GLS-mid; GLS-endo: global longitudinal strain in the endocardial; GLS-mid: global longitudinal strain in the mid-myocardial; GLS-epi: global longitudinal strain in the epicardial; WIS: wash in slope (represents myocardial perfusion velocity); PI: peak intensity (represents myocardial blood volume); AUC: area under of curve.

**Figure 1:**
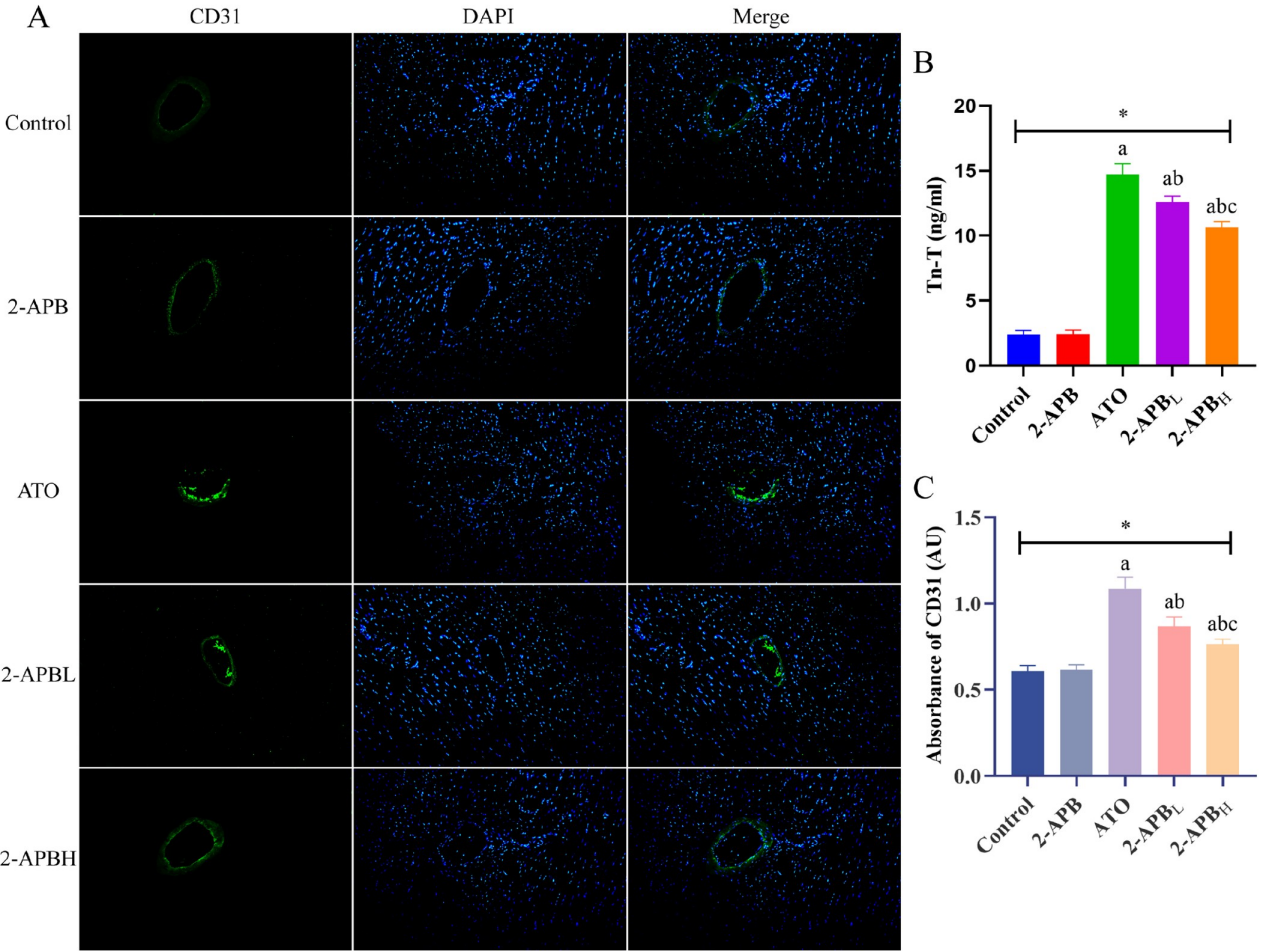
Figure 1 Representative images of CD31 immunofluorescence in the myocardium and Tn-T levels of rats in each group (A) Representative images of CD31 immunofluorescence in the myocardium (×200); blue fluorescence indicates the stained nucleus, and green fluorescence indicates CD31. (B) Tn-T levels in the rats in each group. (C) The absorbance values of CD31 in the rats in each group. * P<0.05: overall comparison between groups; aP<0.05: compared with the control group; bP<0.05: compared with the ATO group; cP<0.05: compared with the low-dose 2-APB group. 2-APBL: low-dose 2-APB group, 2-APBH: high-dose 2-APB group.

MCE was performed on the hearts of rats in each group to determine the relevant parameters of the postwall myocardium that reflect the perfusion of the myocardial microcirculation and, ultimately, the viability of the myocardial tissue [8]. Compared with those in the control group, the wash-in slope (WIS), peak intensity (PI), and area under the curve (AUC) decreased in the ATO group, but the above parameters recovered to different degrees after intervention with different doses of 2-APB ( Table 1). The above results suggested that ATO destroys myocardial microcirculation perfusion in rats and that different doses of 2-APB can restore myocardial microcirculation perfusion to different degrees. In addition, we performed CD31 immunofluorescence staining on the myocardium. One of the markers of vascular endothelial cells is CD31, which is highly expressed at the intercellular junction of vascular endothelial cells and plays an important role in maintaining the integrity of endothelial cell junctions [9]. We found that the capillaries of the myocardial tissue of rats in the control and 2-APB groups were regular and evenly distributed, and the myocardial nuclei were evenly distributed. In the ATO group, a few new capillaries with uneven distribution and different sizes were observed, and a large number of blue nuclei were observed, which was caused by infiltration of endothelial cells, inflammatory cells, fibroblasts, and so on. After intervention with different doses of 2-APB, the expression of CD31 in the myocardium of rats was greater than that in the myocardium of rats in the ATO group, and the infiltration of inflammatory cells and endothelial cells decreased. The results of the CD31 absorbance value showed that the absorbance value of the ATO group was greater than that of the control group, while those of the low-dose and high-dose 2-APB groups were less than that of the ATO group ( Figure 1A,C). The above results suggested that ATO can damage myocardial capillary endothelial cells, that is, destroy myocardial microcirculation perfusion, while 2-APB can reduce the damage caused by ATO to myocardial microcirculation perfusion. The above results indicated that STE combined with MCE can be used to evaluate the cardiotoxicity of ATO, while 2-APB can restore myocardial exercise ability and myocardial microcirculation perfusion in rats.

**Figure 2:**
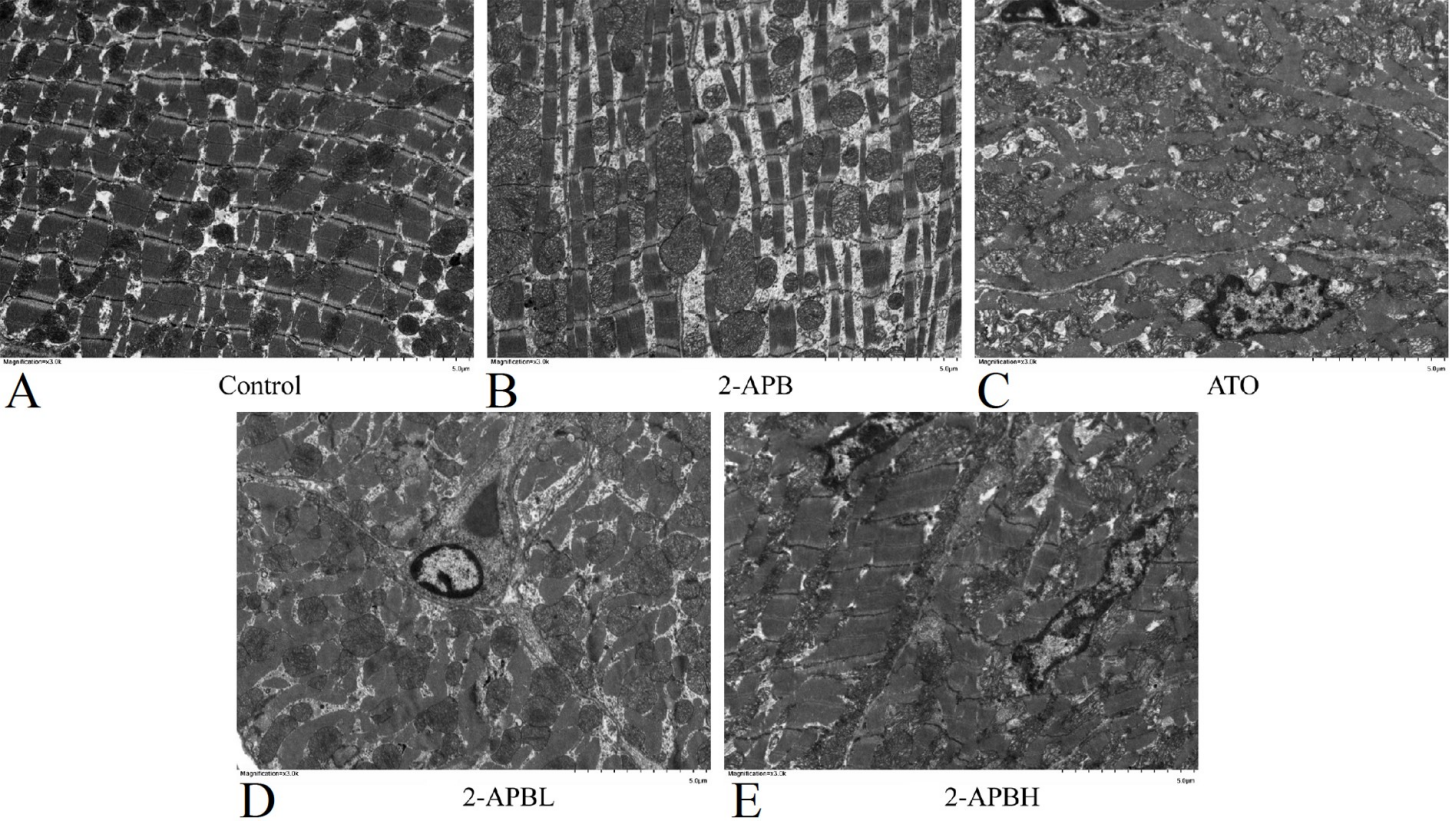
Figure 2 Representative images of the ultrastructure of the myocardium of rats in each group (A) Control group. (B) 2-APB group. (C) ATO group. (D) Low-dose 2-APB group. (E) High-dose 2-APB group. 2-APB L: low-dose 2-APB group, 2-APBH: high-dose 2-APB group. Magnification, ×3000.

To further investigate the effect of 2-APB on the cardiotoxicity induced by ATO, the myofibril structure of the rats in each group was observed under an electron microscope. The results showed that the myocardial myofibrils of rats in the control and 2-APB groups differed in thickness, during which there were abundant mitochondria, and the structures of the A-band, Z-band, and I-band were visible ( Figure 2A,B). The myocardial sarcomere structure of rats in the ATO group was disrupted, which was characterized by the separation of the myocardial myofilament bundle, partial myofilament dissolution, and rupture; the unclear boundary between the A band and I band; the distorted Z band; the disordered arrangement; the deformation and swelling of mitochondria; and even the rupture of some mitochondria ( Figure 2C). After intervention with different doses of 2-APB, the degree of damage to the myocardial sarcomere structure in the rats was alleviated to different degrees ( Figure 2D,E). These results suggested that ATO can damage the structure of myocardial myofibrils, while 2-APB can reduce the degree of myocardial myofibril injury caused by ATO.

**Figure 3:**
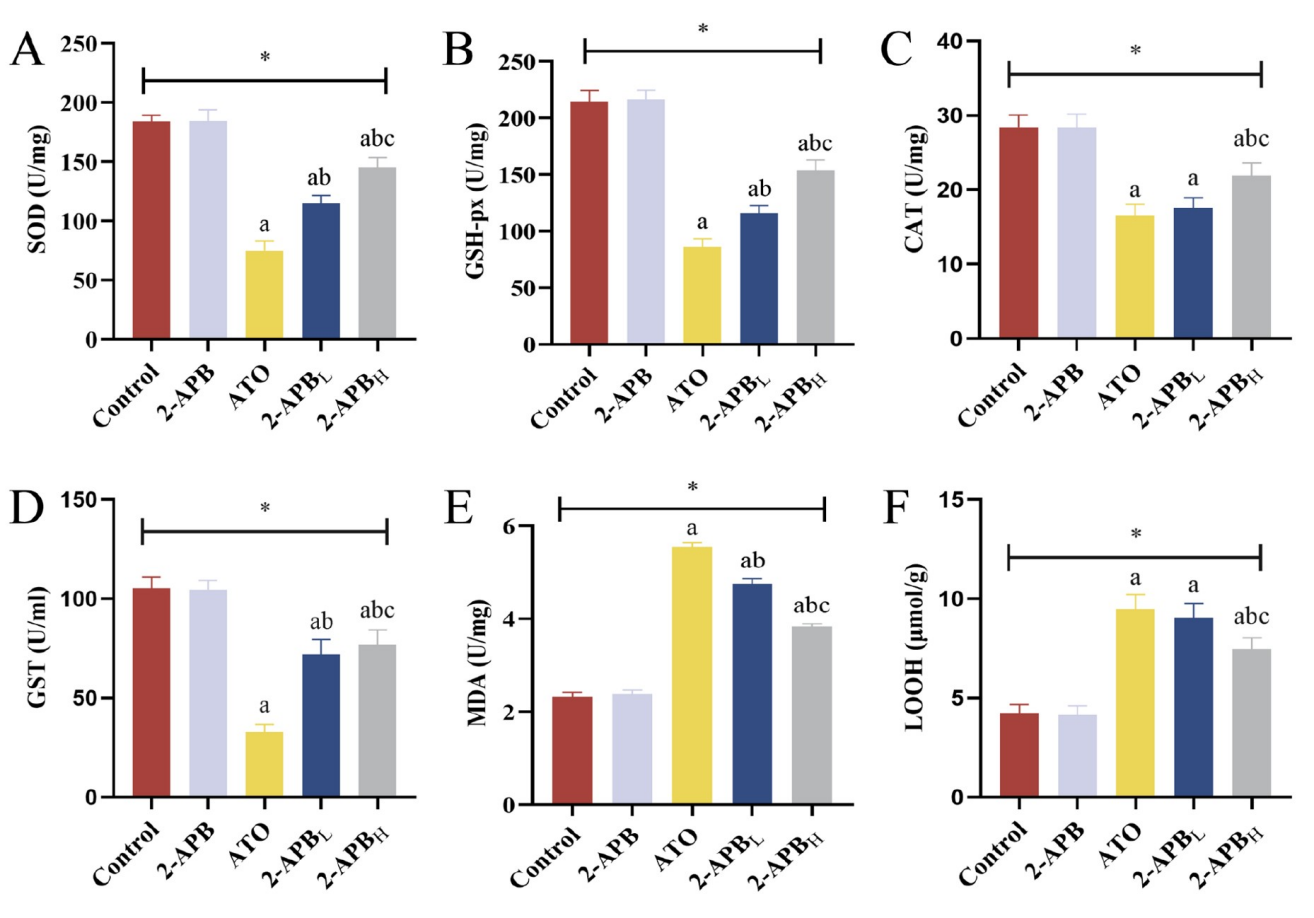
Figure 3 The levels of enzymes and products related to oxidative stress in each group (A) SOD: superoxide dismutase. (B) GSH-px: glutathione peroxidase. (C) CAT: catalase. (D) GST: glutathione S-transferase. (E) MDA: malondialdehyde ratio. (F) LOOH: lipid hydroperoxide. * P<0.05: overall comparison between groups; aP<0.05: compared with the control group; bP<0.05: compared with the ATO group; cP<0.05: compared with the low-dose 2-APB group. 2-APBL: low-dose 2-APB group, 2-APBH: high-dose 2-APB group.

To further confirm the protective effect of 2-APB on the cardiotoxicity induced by ATO. First, we measured the levels of Troponin T (Tn-T) in the serum of rats in each group. Tn-T, a marker of myocardial injury, can reflect the degree of myocardial damage [10]. The results showed that the Tn-T levels in the ATO group were greater than those in the control group, and after intervention with different doses of 2-APB, the Tn-T levels decreased to varying degrees, indicating that 2-APB alleviated the myocardial damage caused by ATO ( Figure 1B). Then, we measured the levels of enzymes and products related to oxidative stress. A previous study showed that arsenic ions in arsenic trioxide can bind with hydroxyl and thiol groups in enzyme protein molecules in the body, thereby reducing antioxidant enzyme activity [11]. SOD is an important antioxidant enzyme in the body that can eliminate harmful substances produced during metabolism, glutathione peroxidase (GSH-px) and catalase (CAT) are important peroxidase-decomposing enzymes in organisms that can protect the structure and function of cell membranes from oxidative damage, and glutathione S-transferase (GST) is a key enzyme in the glutathione binding reaction, with antioxidant and detoxifying effects [12]. When oxidative stress occurs, intracellular lipids are oxidized, producing large amounts of malondialdehyde (MDA) and lipid hydroperoxide (LOOH) [11]. Compared with those in the control group, the SOD, GSH-px, CAT, and GST levels in the ATO group were decreased, while the MDA and LOOH levels were increased. However, after intervention with different doses of 2-APB, the levels of these enzymes decreased to varying degrees, and the levels of MDA and LOOH decreased to varying degrees ( Figure 3). The above results suggested that the cardiotoxic effect of ATO is related to oxidative stress, while 2-APB can alleviate the cardiotoxic effect of ATO by inhibiting oxidative stress. Nevertheless, some limitations exist in this study: there is no clear standard for the dose of 2-APB at present, so the cardioprotective effect of 2-APB needs to be further studied, and we will further study and improve it in follow-up experiments.

In conclusion, 2-APB may reduce the cardiotoxicity of ATO through the overexpression of antioxidant stress, restore myocardial exercise ability and myocardial microcirculation perfusion, and ultimately play a role in myocardial protection. This study provides relevant data and new ideas for the clinical reduction of cardiotoxicity caused by ATO.
